# Supplementary material for: High-frequency gas effusion through nanopores in suspended graphene
Source: Nat Commun. 2020 Nov 27;11:6025. doi: 10.1038/s41467-020-19893-5 (PMC7695699; doi:10.1038/s41467-020-19893-5)
Supplement: Supplementary file 1 — Supplementary Information [file 41467_2020_19893_MOESM1_ESM.pdf]

SUPPLEMENTARY INFORMATION: High-frequency gas effusion through nanopores in suspended graphene

I.E. Rosłoń,[1,2,*] R.J. Dolleman,[1,†] H. Licona,[1] M. Lee,[1] M. Šiškins,[1] H. Lebius,[3]
L. Madauß,[4] M. Schleberger,[4] F.Alijani,[2] H.S.J. van der Zant,[1] and P.G. Steeneken[1,2,‡]
[1]*Kavli Institute of Nanoscience, Delft University of Technology, Lorentzweg 1, 2628 CJ, Delft, The Netherlands*
[2]*Department of Precision and Microsystem Engineering, Faculty 3mE, Delft University of Technology, Mekelweg 2, 2628 CD, Delft, The Netherlands*
[3]*CIMAP/GANIL, CEA-CNRS-ENSICAEN-UCN, blvd Henri Becquerel, F-14070 Caen, France*
[4]*Faculty of Physics and CENIDE, Universität Duisburg-Essen, 47057 Duisburg, Germany*

CONTENTS

Supplementary Note 1: Model derivation 1

Supplementary Note 2: Numerical simulation 3

Supplementary Note 3: Thermal time constant 5

Supplementary Note 4: Measurements on single-layer circular graphene micro drum with nanoperforations 5

Supplementary Note 5: Characterization of mechanical deformations introduced by milling nanoperforations and contamination 7

Supplementary Figure 6: Dataset on graphene drum without perforations 7

Supplementary Figure 7: Additional dataset 8

References 8

SUPPLEMENTARY NOTE 1: MODEL DERIVATION

In this section we derive the model for the complex amplitude of the membrane. The temperature at the center of the membrane $T(t)$ can be approximately described by a first order heat equation:

$$\frac{\mathrm{d}\Delta T}{\mathrm{d}t} = -\frac{\Delta T}{\tau_{\mathrm{th}}} + \frac{\mathcal{P}_{\mathrm{AC}}}{C_{\mathrm{th}}} e^{i\omega t}, \tag{1}$$

where the optothermal laser power $\mathcal{P}_{\mathrm{AC}} e^{i\omega t}$ is absorbed by the graphene membrane and thermal transport towards the substrate is determined by a single thermal time constant $\tau_{\mathrm{th}} = R_{\mathrm{th}} C_{\mathrm{th}}$ corresponding to the product of the membrane's thermal resistance and thermal capacitance.

In the presence of gas, the pressure difference $\Delta P = P - P_{\mathrm{ext}}$ between the cavity pressure P and the ambient pressure P_{ext} can also be described by a differential equation. There are three contributions to the time derivative of the pressure $\mathrm{d}\Delta P/\mathrm{d}t$: gas permeation, motion of the membrane and laser heating of the gas in the cavity.

$$\frac{\mathrm{d}\Delta P}{\mathrm{d}t} = -\frac{\Delta P}{\tau_{\mathrm{gas}}} + \gamma \frac{\mathrm{d}z}{\mathrm{d}t} + \frac{\mathcal{P}_{\mathrm{AC}}}{C_{\mathrm{gas}}} e^{i\omega t} \tag{2}$$

Gas permeation out of the membrane with a time constant τ_{gas} gives a contribution $-\Delta P/\tau_{\mathrm{gas}}$. Compression of the gas by the downward deflection z of the membrane results in a term $\gamma \mathrm{d}z/\mathrm{d}t$, where for small z and cavity depth g,

* E-mail: I.E.Roslon@tudelft.nl
† Present address: Second Institute of Physics, RWTH Aachen University, Otto-Blumenthal-Straße, 52074, Aachen, Germany
‡ E-mail: P.G.Steeneken@tudelft.nl

it can be shown from Boyle's law that $\gamma = \eta P_{ext}/g$, where η is a factor that depends on the deformed shape of the membrane ($\eta = 1$ for a piston like membrane motion). Heating of the gas due to power absorption of the modulated laser can be described by a term $\frac{\mathcal{P}_{\rm AC}}{C_{\rm gas}}e^{i\omega t}$, where $1/C_{\rm gas} = {\rm d}P/{\rm d}U$ is the pressure increase per absorbed laser heat energy U. For a gas at constant volume V, the temperature induced pressure change is given by the ideal gas law as ${\rm d}T/{\rm d}P = \frac{V}{Nk_B}$, where N is the number of gas molecules and k_B is Boltzmann's constant. The temperature change for a certain absorbed amount of heat is given by ${\rm d}U/{\rm d}T = c_v m$, where c_v is the specific heat and m the mass of the gas molecules. Thus it is found that the power induced gas pressure increase is characterized by the constant $C_{\rm gas} = {\rm d}U/{\rm d}T \times {\rm d}T/{\rm d}P = Vmc_v/NK_B$.

A third differential equation is used to describe the mechanics of the membrane, which at low amplitudes experiences a force contribution proportional [1] to the pressure difference $F_P = \beta\Delta P$ and an effective thermal expansion force $\alpha\Delta T$. We approximate the fundamental mode of motion of the center of the membrane by a forced harmonic oscillator with effective mass m_{eff} to obtain:

$$m_{eff}\frac{{\rm d}^2 z}{{\rm d}t^2} + c\frac{{\rm d}z}{{\rm d}t} + kz = \alpha\Delta T + \beta\Delta P. \tag{3}$$

The resulting system of 3 differential equations (1-3) is solved analytically for frequencies below the resonance frequency, where terms proportional to ${\rm d}^2z/{\rm d}t^2$ and ${\rm d}z/{\rm d}t$ can be neglected, to obtain the complex frequency response of the membrane. For frequencies well below the resonance frequency the induced amplitude can be approximated by:

$$z_\omega \approx \alpha\Delta T_\omega + \beta\Delta P_\omega. \tag{4}$$

This can be substituted into equation 2 to arrive at:

$$\frac{d\Delta P}{dt} + \frac{\Delta P}{(1-\beta\gamma)\tau_{\rm gas}} = \frac{\gamma\alpha}{(1-\beta\gamma)}\frac{d\Delta T}{dt} + \frac{\mathcal{P}_{\rm AC}}{(1-\beta\gamma)C_P}e^{i\omega t}. \tag{5}$$

This expression still depends on the temperature ΔT of the membrane. A solution to the temperature ΔT of the membrane following equation 2 in the main text, as found by Dolleman *et al.*, is given by:

$$\Delta T_\omega = \frac{R_{th}\mathcal{P}_{AC}}{i\omega\tau_{th}+1}e^{i\omega t}. \tag{6}$$

This solution is used to arrive at:

$$\frac{d\Delta P}{dt} + \frac{\Delta P}{(1-\beta\gamma)\tau_{\rm gas}} = \frac{\gamma\alpha R_{\rm th}\mathcal{P}_{\rm AC}}{(1-\beta\gamma)}\frac{i\omega e^{i\omega t}}{i\omega\tau_{\rm th}+1} + \frac{\mathcal{P}_{\rm AC}}{(1-\beta\gamma)C_P}e^{i\omega t}. \tag{7}$$

Next, we assume $\gamma = 0$, which holds true for small membrane deflections. We now arrive at:

$$\frac{d\Delta P}{dt} + \frac{\Delta P}{\tau_{\rm gas}} = \frac{\mathcal{P}_{\rm AC}}{C_P}e^{i\omega t}. \tag{8}$$

By solving this differential equation a solution for ΔP is found:

$$\Delta P_\omega = \frac{\tau_{\rm gas}}{C_P}\frac{\mathcal{P}_{AC}}{i\omega\tau_{gas}+1}e^{i\omega t}. \tag{9}$$

By inserting expressions 6 and 9 into formula 4, the complex amplitude z_ω can be obtained:

$$z_\omega e^{i\omega t} = \frac{\alpha R_{\rm th}\mathcal{P}_{\rm AC}}{i\omega\tau_{\rm th}+1}e^{i\omega t} + \frac{\tau_{\rm gas}}{C_P}\frac{\beta\mathcal{P}_{\rm AC}}{i\omega\tau_{\rm gas}+1}e^{i\omega t}. \tag{10}$$

The imaginary part of the complex amplitude is calculated:

$$\mathrm{Im}(z_\omega) = \frac{\alpha\tau_{\rm th}R_{\rm th}\mathcal{P}_{\rm AC}}{1+\omega^2\tau_{\rm th}^2} + \frac{\tau_{\rm gas}}{C_P}\frac{\beta\tau_{\rm gas}\mathcal{P}_{\rm AC}}{1+\omega^2\tau_{\rm gas}^2}. \tag{11}$$

This is the same equation 4 in the main text that is used for fitting, where $a = \alpha R_{\rm th}\mathcal{P}_{\rm AC}$ and $b = \frac{\beta\tau_{\rm gas}\mathcal{P}_{\rm AC}}{C_P}$.

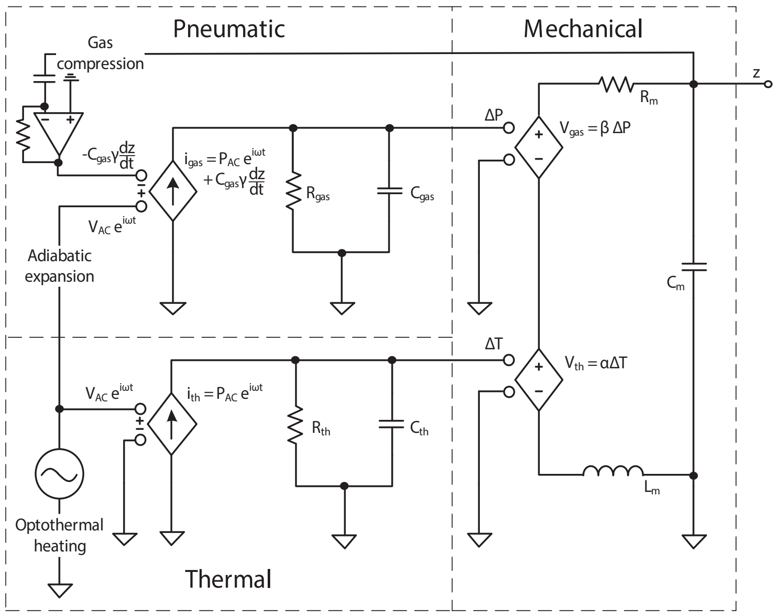

SUPPLEMENTARY FIG. 1. Equivalent electric model for the porous membrane.

SUPPLEMENTARY NOTE 2: NUMERICAL SIMULATION

The system of 3 differential equations (1-3) is numerically simulated using an analogy to the currents running in an electric circuit. The circuit consists of a thermal, a mechanical and a pneumatic domain, as shown in Supplementary figure 1. The domains are discussed one by one. Simulations have been performed using Simulink.

a. Mechanical The mechanical motion of the membrane is represented by a driven damped harmonic oscillator. The equation of motion for the membrane is represented by an RLC circuit in Supplementary figure 1 with a resistor $R_m = c$, an inductor $L_m = m$ and a capacitor $C_m = 1/k$, driven by two voltage controlled voltage sources, $V_{th} = \alpha\Delta T$ and $V_{gas} = \beta\Delta P$. The equation of motion is written next to the expression for the electric potential in this circuit:

$$m\frac{d^2z}{dt^2} + c\frac{dz}{dt} + kz = \alpha\Delta T + \beta\Delta P,$$
$$L_m\frac{d^2q}{dt^2} + R_m\frac{dq}{dt} + \frac{q}{C_m} = V_{\text{th}} + V_{\text{gas}}.$$

Comparison shows that the charge q on the capacitor in this circuit can represent the deflection z of the membrane. In the schematic the voltage over the capacitor, $V_C = \frac{q}{C_m}$, is taken as an output for readout.

b. Thermal The optothermal drive actuating the membrane is represented by an AC voltage source. It controls the voltage controlled current source driving a parallel RC circuit, resembling the thermal flux delivered to the graphene with heat capacity C_{th} and thermal boundary resistance R_{th}. The equation for the membrane temperature is written next to the equation for the currents running through this circuit:

$$\frac{d\Delta T}{dt} + \frac{\Delta T}{\tau_{\text{th}}} = \frac{\mathcal{P}_{\text{AC}}}{C_{\text{th}}}e^{i\omega t},$$
$$C_{\text{th}}\frac{dV_C}{dt} + \frac{V_C}{R_{\text{th}}} = i_{\text{th}}.$$

Comparison shows that the voltage across the capacitor V_C can represent the temperature of the membrane T. Thermal expansion sets the membrane in motion. Therefore, this voltage controls the source driving the circuit in the mechanical domain.

c. Pneumatic The optothermal drive causing adiabatic expansion of the gas is represented by an AC voltage source. Moreover, the movement of the membrane compresses the gas. The voltage over the capacitor in the mechanical domain kz controls a voltage controlled voltage source which is connected to a derivator to change the signal into the effective compression $-C_{\text{gas}}\gamma\frac{dz}{dt}$. A voltage controlled current source drives an RC circuit consisting of a capacitor C_{gas}

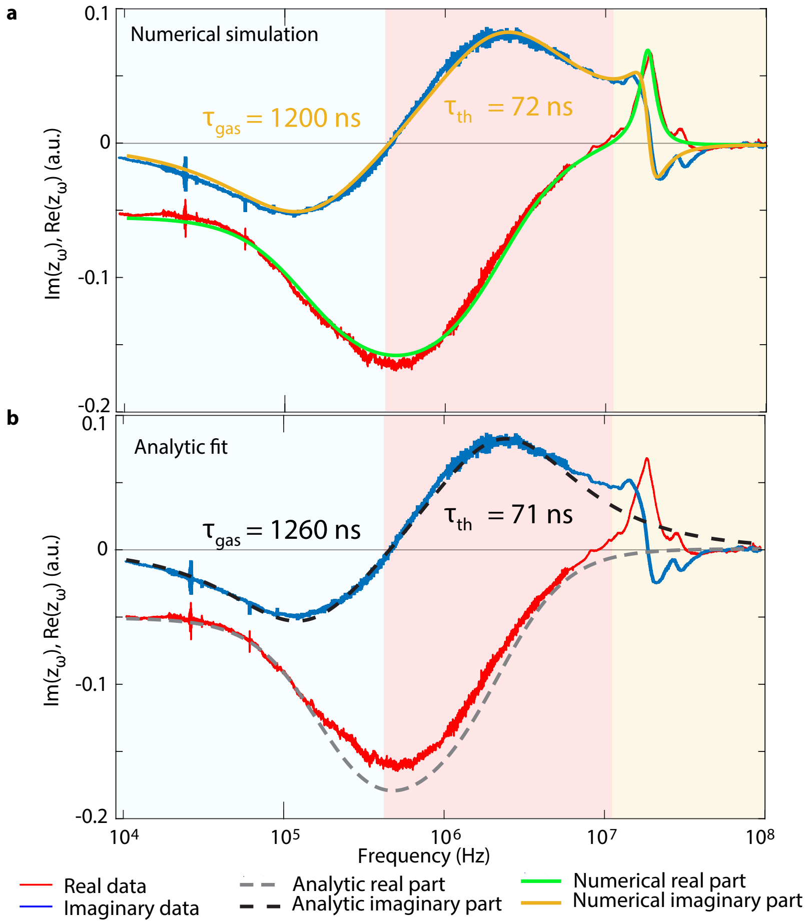

SUPPLEMENTARY FIG. 2. Comparison of the numerical simulation to the analytic formula. a) The governing differential equations (1 - 3) are solved numerically using Simulink (green and yellow), yielding a nearly perfect fit to the measured frequency response curves (blue and red) of the porous membrane including the first resonance peak. b) A fit using the analytic formula (dashed lines), yielding nearly the same fitting parameters as with the numerical simulation.

and a resistor R_{gas} in parallel. This circuit resembles the pressure in the cavity with corresponding effective pressure capacity and permeation resistance. The equation for the pressure in the cavity is written next to the equation for the currents running through this circuit:

$$\frac{d\Delta P}{dt} + \frac{\Delta P}{\tau_{\text{gas}}} = \frac{\mathcal{P}_{\text{AC}}}{C_{\text{gas}}} e^{i\omega t} + \gamma \frac{dz}{dt},$$

$$C_{\text{gas}} \frac{dV_C}{dt} + \frac{V_C}{R_{\text{gas}}} = i_{\text{gas}} + \gamma \frac{dV_z}{dt}.$$

Comparison shows that the voltage across the capacitor V_C can represent the pressure inside the cavity P. The force exerted by the gas sets the membrane in motion. Therefore, this voltage controls the source driving the circuit in the mechanical domain.

The frequency response of a device is numerically simulated. Supplementary figure 2a compares shows data from a device in nitrogen gas with 64 $\times$ 50 nm pores with fitting parameters $\tau_{\text{gas}} = 1200$ ns and $\tau_{\text{th}} = 72$ ns. For comparison, a fit of the analytic solution to the same data is shown in 2b. The analytic solution yields $\tau_{\text{gas}} = 1260$ ns and $\tau_{\text{th}} = 71$ ns. The difference between numerical simulation and analytic fit is 5%, and the numerical simulation includes the primary resonance peak.

SUPPLEMENTARY NOTE 3: THERMAL TIME CONSTANT

It is interesting to also investigate the thermal time constant for the different gases at varying pressures. The presence of gas in the cavity opens a new thermal conduction pathway for the membrane and the thermal time constant is therefore expected to decrease as compared to the vacuum measurement. In view of the small dimensions of the gap between the membrane and the substrate the Knudsen formula is used to calculate the effective thermal conductivity k_{eff} of the gas:

$$\frac{k_{\text{eff}}}{k_0} = \frac{1}{1+2\beta \text{Kn}}. \tag{12}$$

Here, k_0 is the thermal conductivity of the gas and β a constant with a value of about 1.5 that depends on the accommodation coefficient.[2, 3] Both conduction to the substrate and through the gas contribute to the final thermal time-constant:

$$\tau_{\text{th}}^{-1} = \tau_{\text{th,vac}}^{-1} + \frac{\xi k_{\text{eff}}}{\rho c_p h_g d}. \tag{13}$$

Here, h_g, ρ and c_p are the radius, height, density and thermal capacity of the graphene membrane, and d is the cavity depth. A measurement in vacuum is performed to find the thermal equilibration time $\tau_{\text{th,vac}} = 87$ ns, which is comparable to values reported in literature for single layer graphene, [4] suggesting that similar boundary effects are limiting thermal conduction. The constant ξ is a transmission coefficient arising from temperature slip on the solid-gas interface [5]. Supplementary figure 3 shows that the gas indeed provides a new heat conduction pathway, decreasing the thermal time constant as effective thermal conductivity increases. From the data a value of $\xi = 0.17$ is found to fit our experiments.

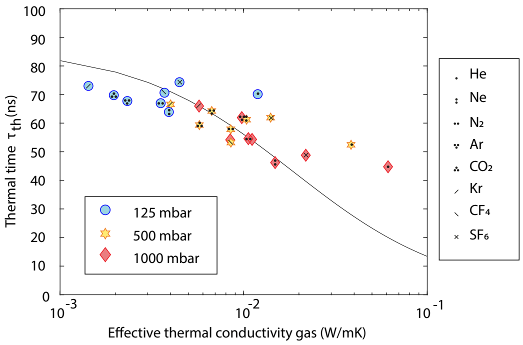

SUPPLEMENTARY FIG. 3. The gas offers a new pathway for heat to escape from the membrane, in consequence lowering the thermal time τ_{th} as the effective thermal conductivity of the gas increases. The black line is a fit to equation 13 with fit parameter $\xi = 0.17$.

Gas sensing can be achieved by observing changes in the thermal time constant in a fashion similar to Pirani gas sensors. In general the gas conducts heat better at higher pressures, and it does so also for molecules with a smaller molecular mass and higher molecular velocity. However, it appears that thermal conductivity of the gases is a less precise route toward gas sensing than the permeation based method discussed in the main text.

SUPPLEMENTARY NOTE 4: MEASUREMENTS ON SINGLE-LAYER CIRCULAR GRAPHENE MICRO DRUM WITH NANOPERFORATIONS

Smaller perforations with sizes below one nanometer could enable molecular sieving and enhance responsivity of these devices. With this purpose, some single layer graphene drums have been exposed to highly energetic ion bombardment with 129Xe^{23+} 0.71 MeV/u, with a flux ranging from $5.09 \cdot 10^7$ to $5.09 \cdot 10^9$ ions per square centimeter at the SME beamline of GANIL (Caen, France). This is similar to the treatment described by Madauß et al.[6] Characterization of the nano indentations on the drum is performed using AFM. The nanopore sizes are distributed normally with mean 14 nm. This experiment is of interest since it shows that our gas sensing principle works using a single layer circular membrane with defects which could potentially lead to applications benefiting from molecular sieving.

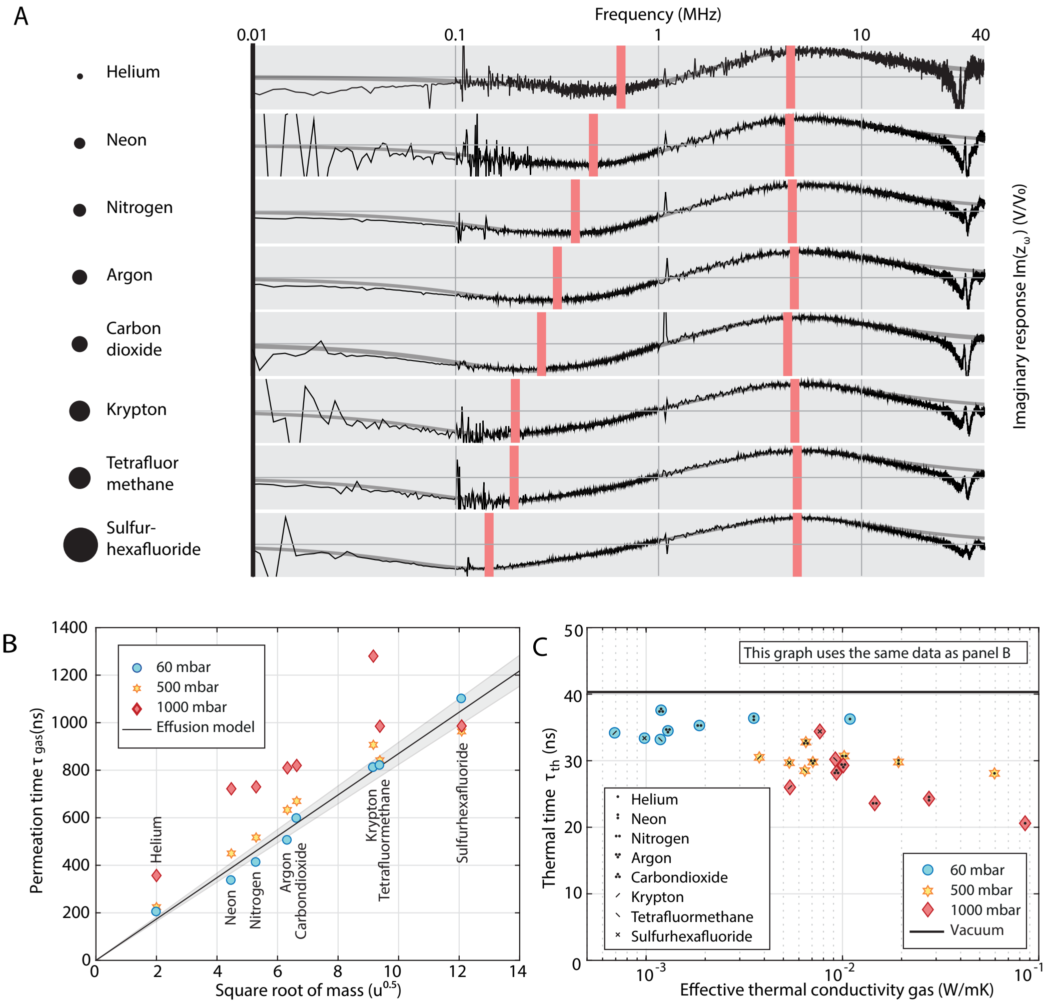

SUPPLEMENTARY FIG. 4. Measurements on a circular SLG drum show the same dip-peak characteristic shape as the dumbbell shaped devices. a) Data (black line) and fit to model (grey line) with the values found for τ_{th} and τ_{gas} indicated by pink marks. b) Permeation time constants follow Graham's law. The black line is a fit to the data at P = 60 mbar. c) Thermal time constants become lower with higher effective thermal conductivity.

SUPPLEMENTARY NOTE 5: CHARACTERIZATION OF MECHANICAL DEFORMATIONS INTRODUCED BY MILLING NANOPERFORATIONS AND CONTAMINATION

Milling pores in the graphene membranes can introduce mechanical deformations that affect the motion and readout of the graphene. Introducing defects directly onto the surface of DLG membrane causes wrinkling with an amplitude of up to 15 nm and reduces signal quality. In the dumbbell geometry wrinkling is reduced by milling pores in the channel rather than directly on the drum, causing only a slight depression in the milled area as can be seen in Supplementary Figure 5. The signal quality from samples perforated in the channel is considerably better compared to drums that are directly perforated. The AFM images also allow to quantify the amount of polymer contamination from the graphene transfer process. In general, contaminants are up to a few nm thick with a few big spots that are less than 15 nm thick. However, we do not expect contaminants to have a large impact on our measurements. The permeation time-constant is measured at frequencies much below the resonance frequency, where the membrane mass does not play a big role, and the permeation time constant is independent on the mass or stiffness of the membrane.

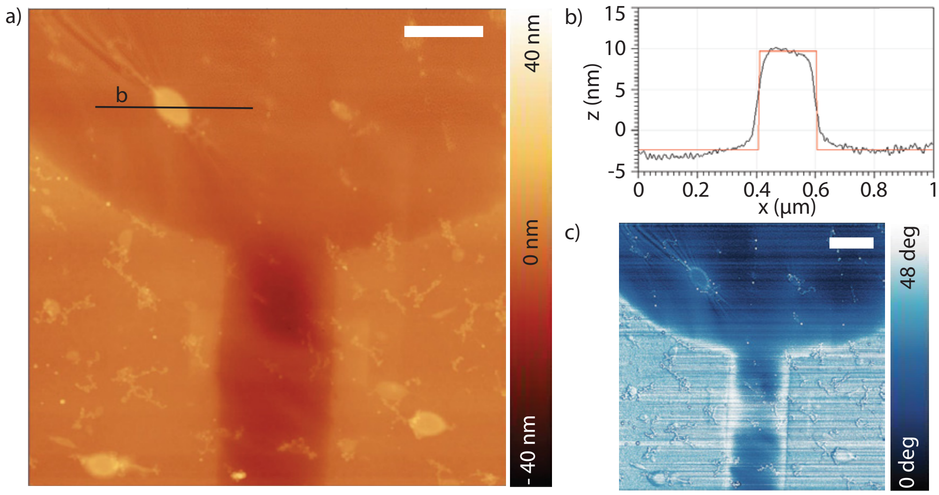

SUPPLEMENTARY FIG. 5. AFM characterization of the samples. a) AFM image of DLG sample with perforations at the channel entrance (n=256 and d=25nm). b) Line scan (black) over a large contamination with fitted height 12 nm (red). c) Phase channel AFM image of the same area as in a. Scalebars are 500 nm long.

SUPPLEMENTARY FIGURE 6: DATASET ON GRAPHENE DRUM WITHOUT PERFORATIONS

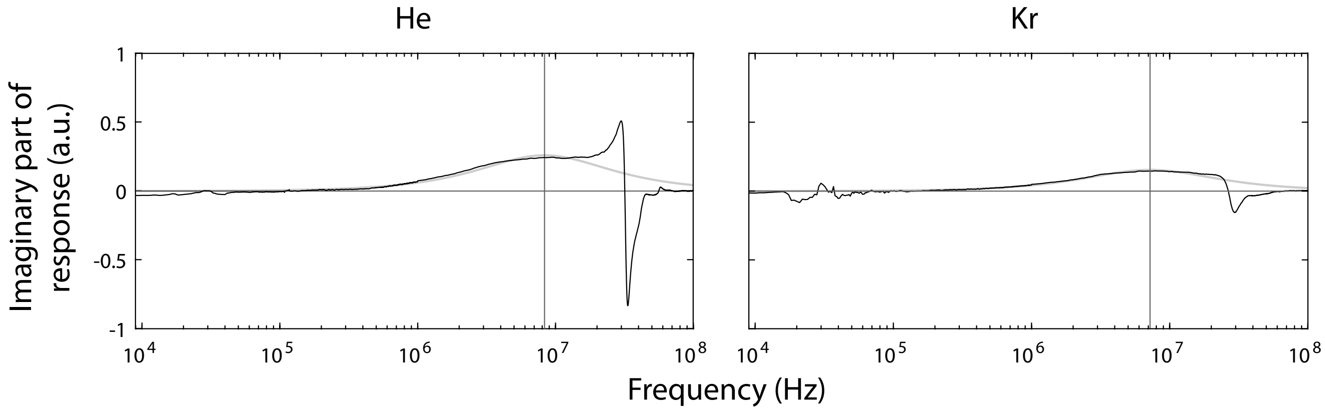

SUPPLEMENTARY FIG. 6. Data (black line) and fit to model (grey line) for He and Kr gas at $P = 125$ mbar measured on an unperforated, pristine sample. Vertical lines indicate the fitted position of the thermal peaks. Without perforations, we do not observe the permeation related dip.

SUPPLEMENTARY FIGURE 7: ADDITIONAL DATASET

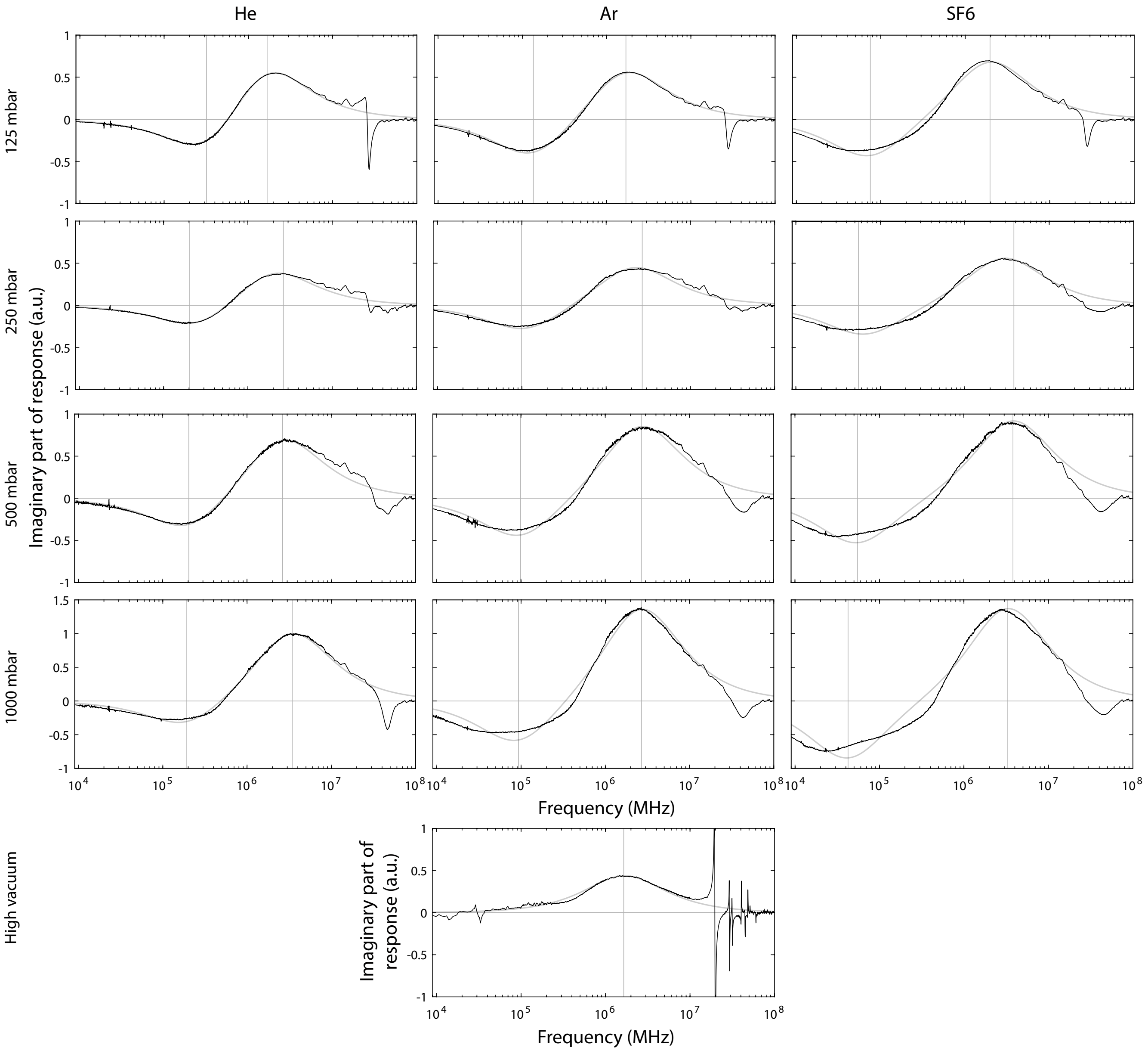

SUPPLEMENTARY FIG. 7. Data (black line) and fit to model (grey line) for 3 gasses at 4 different pressures measured on a sample with 256 pores of diameter 25 nm. Vertical lines indicate the fitted position of the permeation and thermal peaks. For comparison, a measurement on the same device in high vacuum is included. In high vacuum, we do not observe the permeation related dip.

[1] D. Davidovikj, F. Alijani, S. J. Cartamil-Bueno, H. S. van der Zant, M. Amabili, and P. G. Steeneken, Nature Communications **8**, 1253 (2017).

[2] G. Reichenauer, U. Heinemann, and H.-P. Ebert, Colloids and Surfaces A: Physicochemical and Engineering Aspects **300**, 204 (2007).

[3] V. Antonetti, A. Bar Cohen, and A. Bergles, *Fluid Flow Databook* (Genium Publishing, 1981) Chap. 410.2.

[4] R. J. Dolleman, S. Houri, D. Davidovikj, S. J. Cartamil-Bueno, Y. M. Blanter, H. S. van der Zant, and P. G. Steeneken, Physical Review B **96**, 165421 (2017).
[5] D. Singh, X. Guo, A. Alexeenko, J. Y. Murthy, and T. S. Fisher, Journal of Applied Physics **106**, 024314 (2009).
[6] L. Madauß, J. Schumacher, M. Ghosh, O. Ochedowski, J. Meyer, H. Lebius, B. Ban-d'Etat, M. E. Toimil-Molares, C. Trautmann, and R. Lammertink, Nanoscale **9**, 10487 (2017).
